# *catena*-Poly[[bis­(dicyanamido-κ*N* ^1^)cobalt(II)]bis­{μ-1,2-bis­[(1,2,4-triazol-1-yl)meth­yl]benzene-κ^2^ *N* ^4^:*N* ^4′^}]

**DOI:** 10.1107/S1600536814012331

**Published:** 2014-06-04

**Authors:** Jixia Zhang, Xiaoping Shen

**Affiliations:** a School of Chemistry and Chemical Engineering, Jiangsu University, Zhenjiang 212013, People’s Republic of China

## Abstract

In the title complex, [Co(C_2_N_3_)_2_(C_12_H_12_N_6_)_2_]_*n*_ the Co^II^ atom lies on a centre of symmetry and displays a slightly distorted octa­hedral coordination geometry. The 1,2-bis­[(1,2,4-triazol-1-yl)meth­yl]benzene ligands link adjacent metal atoms into polymeric chains parallel to the *c* axis, forming centrosymmetric 26-membered metallamacrocycles. The conformation of the metallamacrocycles is stabilized by pairs of C—H⋯N hydrogen bonds. The dihedral angles formed by the planes of the triazole rings with those of the benzene ring are 79.4 (2) and 79.1 (2)°. In the crystal, the chains inter­act through C—H⋯N hydrogen bonds, forming a three-dimensional network.

## Related literature   

For background to transition metal complexes of 1,2,4-triazole derivatives, see: Haasnoot (2000 ▶); Cui *et al.* (2012 ▶); Han *et al.* (2012 ▶); Wang *et al.* (2012 ▶).
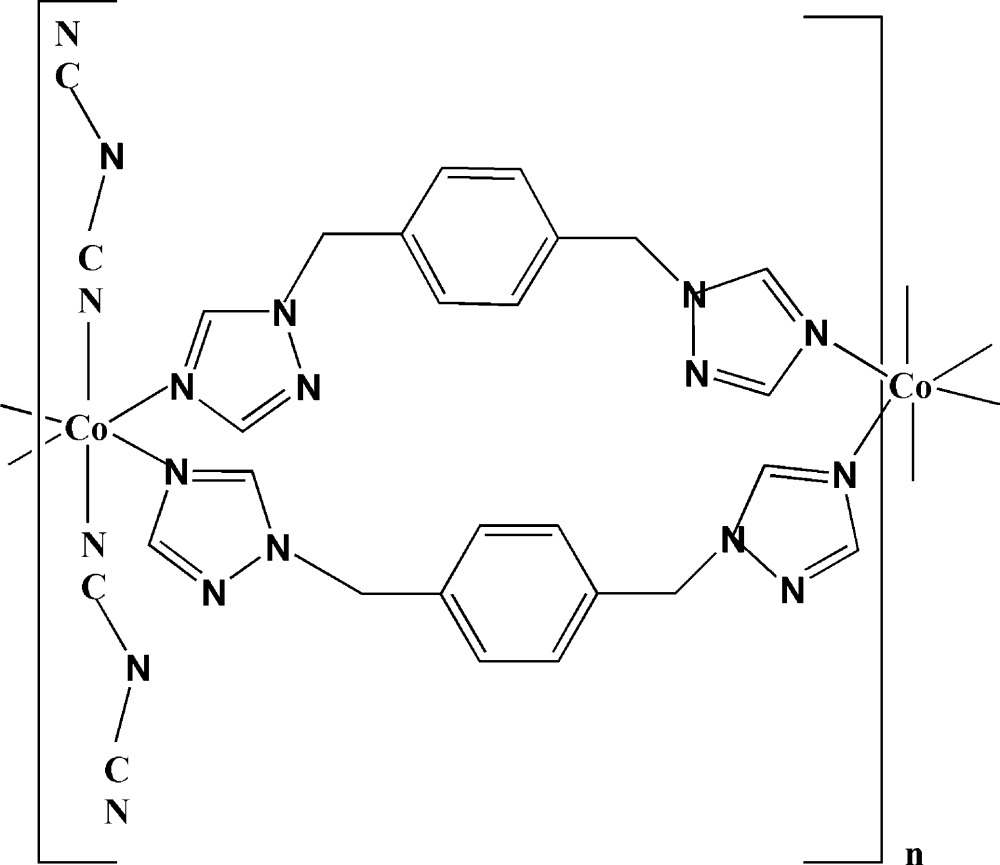


## Experimental   

### 

#### Crystal data  

[Co(C_2_N_3_)_2_(C_12_H_12_N_6_)_2_]
*M* *_r_* = 671.58
Triclinic, 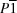

*a* = 8.517 (2) Å
*b* = 9.092 (2) Å
*c* = 9.622 (3) Å
α = 93.984 (7)°
β = 95.015 (7)°
γ = 97.587 (7)°
*V* = 733.2 (3) Å^3^
*Z* = 1
Mo *K*α radiation
μ = 0.64 mm^−1^
*T* = 293 K
0.30 × 0.20 × 0.18 mm

#### Data collection  

Rigaku Mercury CCD diffractometer
Absorption correction: multi-scan (*REQAB*; Jacobson, 1998 ▶) *T*_min_ = 0.831, *T*_max_ = 0.893
7244 measured reflections
2658 independent reflections
2118 reflections with *I* > 2σ(*I*)
*R*_int_ = 0.041

#### Refinement  

*R*[*F* ^2^ > 2σ(*F* ^2^)] = 0.049
*wR*(*F* ^2^) = 0.111
*S* = 1.05
2658 reflections
214 parameters
H-atom parameters constrained
Δρ_max_ = 0.22 e Å^−3^
Δρ_min_ = −0.29 e Å^−3^

### 

Data collection: *CrystalClear* (Rigaku, 2000 ▶); cell refinement: *CrystalClear*; data reduction: *CrystalClear*; program(s) used to solve structure: *SHELXS97* (Sheldrick, 2008 ▶); program(s) used to refine structure: *SHELXL97* (Sheldrick, 2008 ▶); molecular graphics: *SHELXTL* (Sheldrick, 2008 ▶); software used to prepare material for publication: *SHELXTL*.

## Supplementary Material

Crystal structure: contains datablock(s) I, New_Global_Publ_Block. DOI: 10.1107/S1600536814012331/rz5127sup1.cif

Structure factors: contains datablock(s) I. DOI: 10.1107/S1600536814012331/rz5127Isup2.hkl

CCDC reference: 1005518

Additional supporting information:  crystallographic information; 3D view; checkCIF report

## Figures and Tables

**Table 1:** Hydrogen-bond geometry (Å, °)

*D*—H⋯*A*	*D*—H	H⋯*A*	*D*⋯*A*	*D*—H⋯*A*
C9—H9*A*⋯N9^i^	0.93	2.54	3.308 (4)	140
C11—H11*A*⋯N5^ii^	0.93	2.56	3.217 (5)	128
C12—H12*A*⋯N9^iii^	0.93	2.48	3.286 (4)	145

Symmetry codes: (i) 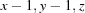; (ii) 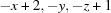; (iii) 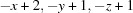.

**Fig. 1.**
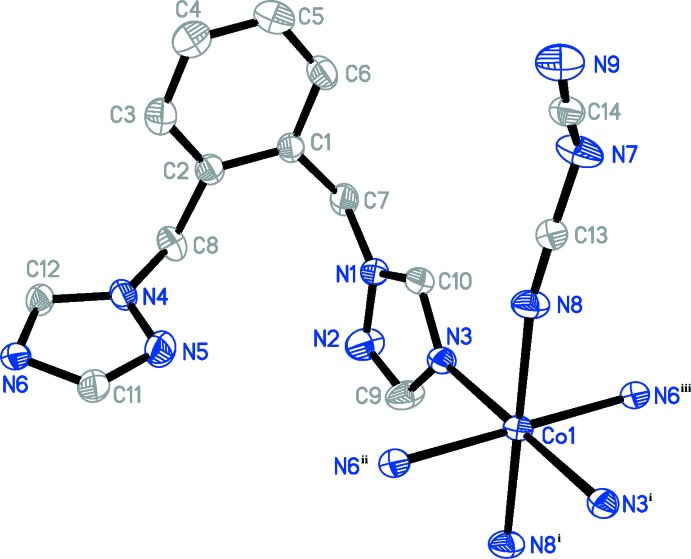
The asymmetric unit of the title compound with displacement ellipsoids drawn at the 30% probability level. Symmetry codes: (i) -x + 2, -y, -z; (ii) -x + 2, -y, -z + 1; (iii) x, y, z - 1]. Hydrogen atoms are omitted for clarity.

**Fig. 2.**
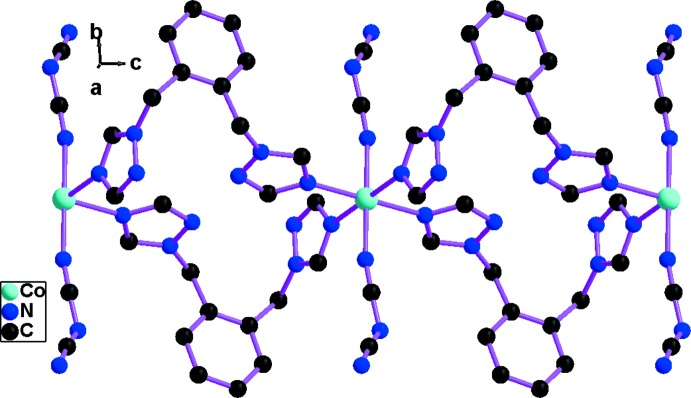
The polymeric one-dimensional chain in the title compound. Hydrogen atoms are omitted for clarity.

## supplementary crystallographic information

### S1. Comment

A large number of mononuclear, oligonuclear and polynuclear transition metal complexes of 1,2,4-triazole derivatives have been synthesized and characterized due to their magnetic properties and novel topologies (Haasnoot, 2000; Cui *et al.*, Han *et al.*, and Wang *et al.*, 2012). As a contribution to this field, we report herein the crystal structure of the title compound.

The asymmetric unit of the title compound is shown in Fig. 1. Each cobalt(II) atom lies on a centre of symmetry and displays a slightly distorted octahedral coordination geometry, provided by four nitrogen atoms from four symmetry-related obtz ligands forming the equatorial plane and by two nitrogen atoms from two dca anions at the apices (obtz = 1,2-bis(1,2,4-triazol-1-ylmethyl)benzene, dca = dicyanamide). Two centrosymmetrically-related obtz ligands link adjacent cobalt(II) atoms to form 22-membered metallamacrocycles, which are connected into one-dimensional chains running parallel to the *c* axis (Fig. 2). The Co···Co separations within the chain are equal to the *c*-axis translation (9.622 (3) Å). The obtz ligands exhibit a *gauche* conformation. The triazole rings form a dihedral angle of 80.5 (2)° and are inclined by 79.4 (2) and 79.1 (2)° with respect to the benzene ring. The conformation of the metallamacrocycles is enforced by pairs of C—H···N hydrogen bonds (Table 1). In the crystal, chains interact through C—H···N hydrogen bonds (Table 1) to form a three-dimensional network.

### S2. Experimental

A 20 ml H_2_O/MeOH solution (1:1 *v*/*v*) of Co(NO_3_)_2_^.^6H_2_O (0.5 mmol) was added to one leg of an "H-shaped" tube, and a 20 ml H_2_O/MeOH (1:1 *v*/*v*) solution of obtz (1.0 mmol) and Na[N(CN)_2_] (1.0 mmol) was added to the other leg of the tube. After two weeks, well shaped single crystals were obtained. Yield: 61%. Found: C, 50.03; H, 3.54; N, 37.49. Calcd. for C_28_H_24_CoN_18_ (I): C, 50.08; H, 3.60; N, 37.55%.

### S3. Refinement

H atoms were placed in idealized positions and refined as riding, with C—H distances of 0.93 (triazole and benzene) and 0.97 Å (methylene), and with *U*_iso_(H) = 1.2 *U*_eq_(C).

### Crystal data

**Table:**

[Co(C_2_N_3_)_2_(C_12_H_12_N_6_)_2_]	*Z* = 1
*M**_r_* = 671.58	*F*(000) = 345
Triclinic, *P*1	*D*_x_ = 1.521 Mg m^−^^3^
Hall symbol: -P 1	Mo *K*α radiation, λ = 0.71070 Å
*a* = 8.517 (2) Å	Cell parameters from 2443 reflections
*b* = 9.092 (2) Å	θ = 3.1–25.4°
*c* = 9.622 (3) Å	µ = 0.64 mm^−^^1^
α = 93.984 (7)°	*T* = 293 K
β = 95.015 (7)°	Block, orange
γ = 97.587 (7)°	0.30 × 0.20 × 0.18 mm
*V* = 733.2 (3) Å^3^	

### Data collection

**Table:**

Rigaku Mercury CCD diffractometer	2658 independent reflections
Radiation source: fine-focus sealed tube	2118 reflections with *I* > 2σ(*I*)
Graphite monochromator	*R*_int_ = 0.041
Detector resolution: 7.31 pixels mm^-1^	θ_max_ = 25.3°, θ_min_ = 3.1°
ω scans	*h* = −9→10
Absorption correction: multi-scan (*REQAB*; Jacobson, 1998)	*k* = −10→10
*T*_min_ = 0.831, *T*_max_ = 0.893	*l* = −11→9
7244 measured reflections	

### Refinement

**Table:**

Refinement on *F*^2^	Primary atom site location: structure-invariant direct methods
Least-squares matrix: full	Secondary atom site location: difference Fourier map
*R*[*F*^2^ > 2σ(*F*^2^)] = 0.049	Hydrogen site location: inferred from neighbouring sites
*wR*(*F*^2^) = 0.111	H-atom parameters constrained
*S* = 1.05	*w* = 1/[σ^2^(*F*_o_^2^) + (0.0447*P*)^2^ + 0.3399*P*] where *P* = (*F*_o_^2^ + 2*F*_c_^2^)/3
2658 reflections	(Δ/σ)_max_ < 0.001
214 parameters	Δρ_max_ = 0.22 e Å^−^^3^
0 restraints	Δρ_min_ = −0.29 e Å^−^^3^

### Special details

**Table:**

Geometry. All e.s.d.'s (except the e.s.d. in the dihedral angle between two l.s. planes) are estimated using the full covariance matrix. The cell e.s.d.'s are taken into account individually in the estimation of e.s.d.'s in distances, angles and torsion angles; correlations between e.s.d.'s in cell parameters are only used when they are defined by crystal symmetry. An approximate (isotropic) treatment of cell e.s.d.'s is used for estimating e.s.d.'s involving l.s. planes.
Refinement. Refinement of *F*^2^ against ALL reflections. The weighted *R*-factor *wR* and goodness of fit *S* are based on *F*^2^, conventional *R*-factors *R* are based on *F*, with *F* set to zero for negative *F*^2^. The threshold expression of *F*^2^ > σ(*F*^2^) is used only for calculating *R*-factors(gt) *etc*. and is not relevant to the choice of reflections for refinement. *R*-factors based on *F*^2^ are statistically about twice as large as those based on *F*, and *R*- factors based on ALL data will be even larger.

### Fractional atomic coordinates and isotropic or equivalent isotropic displacement parameters (Å^2^)

**Table:**

	*x*	*y*	*z*	*U*_iso_*/*U*_eq_	
Co1	1.0000	0.0000	0.0000	0.0318 (2)	
N1	0.6352 (3)	0.1812 (3)	0.2157 (2)	0.0372 (6)	
N2	0.5728 (4)	0.0354 (3)	0.2084 (3)	0.0596 (8)	
N3	0.7995 (3)	0.0625 (3)	0.1044 (2)	0.0344 (6)	
N4	0.7413 (3)	0.1313 (3)	0.6393 (2)	0.0399 (6)	
N5	0.8430 (4)	0.0608 (3)	0.5692 (3)	0.0589 (8)	
N6	0.8898 (3)	0.0428 (3)	0.8003 (2)	0.0358 (6)	
N7	1.1984 (3)	0.4878 (3)	0.0020 (3)	0.0530 (8)	
N8	1.1042 (3)	0.2262 (3)	0.0262 (3)	0.0433 (7)	
N9	1.4468 (3)	0.6425 (3)	0.0931 (3)	0.0545 (8)	
C1	0.6521 (4)	0.3991 (3)	0.3874 (3)	0.0397 (7)	
C2	0.6915 (4)	0.3559 (4)	0.5211 (3)	0.0408 (8)	
C3	0.7892 (4)	0.4559 (4)	0.6162 (4)	0.0562 (9)	
H3A	0.8178	0.4273	0.7048	0.067*	
C4	0.8452 (5)	0.5969 (4)	0.5828 (4)	0.0673 (11)	
H4A	0.9114	0.6621	0.6483	0.081*	
C5	0.8034 (5)	0.6410 (4)	0.4529 (4)	0.0695 (11)	
H5A	0.8385	0.7370	0.4308	0.083*	
C6	0.7093 (5)	0.5422 (4)	0.3556 (4)	0.0562 (9)	
H6A	0.6834	0.5715	0.2668	0.067*	
C7	0.5494 (4)	0.2970 (4)	0.2755 (3)	0.0463 (8)	
H7A	0.4564	0.2499	0.3151	0.056*	
H7B	0.5130	0.3552	0.2015	0.056*	
C8	0.6221 (4)	0.2075 (4)	0.5666 (3)	0.0469 (8)	
H8A	0.5403	0.2229	0.6281	0.056*	
H8B	0.5722	0.1446	0.4849	0.056*	
C9	0.6753 (4)	−0.0309 (4)	0.1403 (4)	0.0540 (9)	
H9A	0.6631	−0.1333	0.1188	0.065*	
C10	0.7693 (3)	0.1939 (3)	0.1547 (3)	0.0348 (7)	
H10A	0.8339	0.2835	0.1482	0.042*	
C11	0.9294 (4)	0.0098 (4)	0.6696 (3)	0.0539 (9)	
H11A	1.0116	−0.0449	0.6525	0.065*	
C12	0.7701 (4)	0.1187 (3)	0.7761 (3)	0.0401 (7)	
H12A	0.7136	0.1580	0.8448	0.048*	
C13	1.1548 (3)	0.3483 (3)	0.0180 (3)	0.0356 (7)	
C14	1.3340 (4)	0.5617 (3)	0.0535 (3)	0.0396 (7)	

### Atomic displacement parameters (Å^2^)

**Table:**

	*U*^11^	*U*^22^	*U*^33^	*U*^12^	*U*^13^	*U*^23^
Co1	0.0361 (3)	0.0240 (3)	0.0352 (3)	0.0040 (2)	0.0037 (2)	0.0028 (2)
N1	0.0416 (15)	0.0357 (15)	0.0358 (14)	0.0106 (12)	0.0040 (12)	0.0037 (11)
N2	0.0564 (19)	0.0465 (18)	0.077 (2)	−0.0006 (15)	0.0282 (16)	−0.0005 (16)
N3	0.0371 (14)	0.0302 (14)	0.0366 (13)	0.0042 (11)	0.0064 (11)	0.0045 (11)
N4	0.0457 (15)	0.0430 (15)	0.0340 (14)	0.0121 (12)	0.0061 (12)	0.0100 (12)
N5	0.078 (2)	0.066 (2)	0.0397 (16)	0.0324 (17)	0.0117 (15)	0.0035 (15)
N6	0.0418 (15)	0.0306 (14)	0.0362 (14)	0.0077 (11)	0.0069 (11)	0.0018 (11)
N7	0.0491 (17)	0.0319 (16)	0.074 (2)	−0.0018 (13)	−0.0094 (15)	0.0137 (14)
N8	0.0464 (16)	0.0276 (15)	0.0554 (17)	0.0016 (12)	0.0081 (13)	0.0027 (12)
N9	0.0522 (18)	0.0388 (17)	0.070 (2)	0.0013 (15)	0.0032 (16)	−0.0001 (15)
C1	0.0480 (19)	0.0432 (19)	0.0336 (16)	0.0219 (15)	0.0086 (14)	0.0063 (14)
C2	0.0428 (18)	0.0438 (19)	0.0388 (17)	0.0132 (15)	0.0090 (14)	0.0051 (15)
C3	0.067 (2)	0.057 (2)	0.043 (2)	0.0051 (19)	0.0021 (18)	0.0044 (18)
C4	0.080 (3)	0.056 (2)	0.061 (3)	0.001 (2)	−0.001 (2)	−0.004 (2)
C5	0.091 (3)	0.045 (2)	0.072 (3)	0.002 (2)	0.011 (2)	0.012 (2)
C6	0.077 (3)	0.053 (2)	0.046 (2)	0.022 (2)	0.0127 (19)	0.0172 (18)
C7	0.051 (2)	0.056 (2)	0.0377 (17)	0.0278 (17)	0.0034 (15)	0.0071 (16)
C8	0.0436 (19)	0.055 (2)	0.0440 (19)	0.0090 (16)	0.0008 (15)	0.0165 (16)
C9	0.054 (2)	0.0355 (19)	0.073 (2)	−0.0015 (16)	0.0240 (19)	−0.0050 (17)
C10	0.0358 (17)	0.0304 (16)	0.0385 (16)	0.0042 (13)	0.0046 (14)	0.0050 (13)
C11	0.072 (2)	0.055 (2)	0.0413 (19)	0.0341 (19)	0.0077 (17)	0.0024 (17)
C12	0.0437 (19)	0.0464 (19)	0.0329 (16)	0.0124 (15)	0.0099 (14)	0.0031 (14)
C13	0.0378 (17)	0.0336 (18)	0.0356 (17)	0.0055 (14)	0.0047 (13)	0.0023 (14)
C14	0.049 (2)	0.0287 (17)	0.0442 (18)	0.0092 (16)	0.0119 (16)	0.0059 (14)

### Geometric parameters (Å, º)

**Table:**

Co1—N8^i^	2.118 (3)	N9—C14	1.148 (4)
Co1—N8	2.118 (3)	C1—C6	1.392 (5)
Co1—N6^ii^	2.147 (2)	C1—C2	1.397 (4)
Co1—N6^iii^	2.147 (2)	C1—C7	1.506 (4)
Co1—N3	2.174 (2)	C2—C3	1.381 (4)
Co1—N3^i^	2.174 (2)	C2—C8	1.510 (4)
N1—C10	1.325 (4)	C3—C4	1.377 (5)
N1—N2	1.356 (4)	C3—H3A	0.9300
N1—C7	1.472 (4)	C4—C5	1.370 (5)
N2—C9	1.319 (4)	C4—H4A	0.9300
N3—C10	1.322 (4)	C5—C6	1.374 (5)
N3—C9	1.352 (4)	C5—H5A	0.9300
N4—C12	1.333 (4)	C6—H6A	0.9300
N4—N5	1.344 (4)	C7—H7A	0.9700
N4—C8	1.459 (4)	C7—H7B	0.9700
N5—C11	1.311 (4)	C8—H8A	0.9700
N6—C12	1.318 (4)	C8—H8B	0.9700
N6—C11	1.354 (4)	C9—H9A	0.9300
N6—Co1^iv^	2.147 (2)	C10—H10A	0.9300
N7—C14	1.296 (4)	C11—H11A	0.9300
N7—C13	1.297 (4)	C12—H12A	0.9300
N8—C13	1.147 (4)		
			
N8^i^—Co1—N8	180.00 (6)	C4—C3—H3A	119.3
N8^i^—Co1—N6^ii^	88.36 (9)	C2—C3—H3A	119.3
N8—Co1—N6^ii^	91.64 (9)	C5—C4—C3	120.0 (4)
N8^i^—Co1—N6^iii^	91.64 (9)	C5—C4—H4A	120.0
N8—Co1—N6^iii^	88.36 (9)	C3—C4—H4A	120.0
N6^ii^—Co1—N6^iii^	180.00 (13)	C4—C5—C6	119.5 (4)
N8^i^—Co1—N3	91.36 (9)	C4—C5—H5A	120.3
N8—Co1—N3	88.64 (9)	C6—C5—H5A	120.3
N6^ii^—Co1—N3	88.73 (9)	C5—C6—C1	121.3 (3)
N6^iii^—Co1—N3	91.27 (9)	C5—C6—H6A	119.3
N8^i^—Co1—N3^i^	88.64 (9)	C1—C6—H6A	119.3
N8—Co1—N3^i^	91.36 (9)	N1—C7—C1	112.1 (2)
N6^ii^—Co1—N3^i^	91.27 (9)	N1—C7—H7A	109.2
N6^iii^—Co1—N3^i^	88.73 (9)	C1—C7—H7A	109.2
N3—Co1—N3^i^	180.00 (12)	N1—C7—H7B	109.2
C10—N1—N2	109.0 (2)	C1—C7—H7B	109.2
C10—N1—C7	130.1 (3)	H7A—C7—H7B	107.9
N2—N1—C7	120.8 (3)	N4—C8—C2	112.7 (3)
C9—N2—N1	103.0 (3)	N4—C8—H8A	109.0
C10—N3—C9	102.4 (3)	C2—C8—H8A	109.0
C10—N3—Co1	131.0 (2)	N4—C8—H8B	109.0
C9—N3—Co1	126.5 (2)	C2—C8—H8B	109.0
C12—N4—N5	109.6 (2)	H8A—C8—H8B	107.8
C12—N4—C8	129.0 (3)	N2—C9—N3	114.4 (3)
N5—N4—C8	121.4 (2)	N2—C9—H9A	122.8
C11—N5—N4	102.8 (3)	N3—C9—H9A	122.8
C12—N6—C11	102.3 (3)	N3—C10—N1	111.2 (3)
C12—N6—Co1^iv^	127.4 (2)	N3—C10—H10A	124.4
C11—N6—Co1^iv^	130.2 (2)	N1—C10—H10A	124.4
C14—N7—C13	124.1 (3)	N5—C11—N6	114.8 (3)
C13—N8—Co1	169.2 (3)	N5—C11—H11A	122.6
C6—C1—C2	119.0 (3)	N6—C11—H11A	122.6
C6—C1—C7	118.3 (3)	N6—C12—N4	110.5 (3)
C2—C1—C7	122.7 (3)	N6—C12—H12A	124.7
C3—C2—C1	118.7 (3)	N4—C12—H12A	124.7
C3—C2—C8	119.4 (3)	N8—C13—N7	174.2 (3)
C1—C2—C8	121.8 (3)	N9—C14—N7	171.4 (3)
C4—C3—C2	121.5 (3)		
			
C10—N1—N2—C9	−0.8 (4)	C2—C1—C6—C5	0.0 (5)
C7—N1—N2—C9	175.3 (3)	C7—C1—C6—C5	179.9 (3)
N8^i^—Co1—N3—C10	−178.4 (3)	C10—N1—C7—C1	−56.9 (4)
N8—Co1—N3—C10	1.6 (3)	N2—N1—C7—C1	128.0 (3)
N6^ii^—Co1—N3—C10	93.2 (3)	C6—C1—C7—N1	105.8 (3)
N6^iii^—Co1—N3—C10	−86.8 (3)	C2—C1—C7—N1	−74.3 (4)
N8^i^—Co1—N3—C9	5.4 (3)	C12—N4—C8—C2	102.7 (4)
N8—Co1—N3—C9	−174.6 (3)	N5—N4—C8—C2	−77.0 (4)
N6^ii^—Co1—N3—C9	−83.0 (3)	C3—C2—C8—N4	−49.1 (4)
N6^iii^—Co1—N3—C9	97.0 (3)	C1—C2—C8—N4	134.9 (3)
C12—N4—N5—C11	−0.4 (4)	N1—N2—C9—N3	0.4 (4)
C8—N4—N5—C11	179.3 (3)	C10—N3—C9—N2	0.1 (4)
N6^ii^—Co1—N8—C13	162.9 (13)	Co1—N3—C9—N2	177.2 (2)
N6^iii^—Co1—N8—C13	−17.1 (13)	C9—N3—C10—N1	−0.6 (3)
N3—Co1—N8—C13	−108.5 (13)	Co1—N3—C10—N1	−177.47 (18)
N3^i^—Co1—N8—C13	71.5 (13)	N2—N1—C10—N3	0.9 (3)
C6—C1—C2—C3	−1.5 (5)	C7—N1—C10—N3	−174.6 (3)
C7—C1—C2—C3	178.6 (3)	N4—N5—C11—N6	0.1 (4)
C6—C1—C2—C8	174.6 (3)	C12—N6—C11—N5	0.3 (4)
C7—C1—C2—C8	−5.3 (5)	Co1^iv^—N6—C11—N5	−175.5 (2)
C1—C2—C3—C4	1.3 (5)	C11—N6—C12—N4	−0.6 (3)
C8—C2—C3—C4	−174.9 (3)	Co1^iv^—N6—C12—N4	175.45 (18)
C2—C3—C4—C5	0.4 (6)	N5—N4—C12—N6	0.7 (4)
C3—C4—C5—C6	−1.9 (6)	C8—N4—C12—N6	−179.0 (3)
C4—C5—C6—C1	1.7 (6)		

Symmetry codes: (i) −*x*+2, −*y*, −*z*; (ii) −*x*+2, −*y*, −*z*+1; (iii) *x*, *y*, *z*−1; (iv) *x*, *y*, *z*+1.

### Hydrogen-bond geometry (Å, º)

**Table:**

*D*—H···*A*	*D*—H	H···*A*	*D*···*A*	*D*—H···*A*
C9—H9*A*···N9^v^	0.93	2.54	3.308 (4)	140
C11—H11*A*···N5^ii^	0.93	2.56	3.217 (5)	128
C12—H12*A*···N9^vi^	0.93	2.48	3.286 (4)	145

Symmetry codes: (ii) −*x*+2, −*y*, −*z*+1; (v) *x*−1, *y*−1, *z*; (vi) −*x*+2, −*y*+1, −*z*+1.
